# Apolar Extracts of St. John’s Wort Alleviate the Effects of β-Amyloid Toxicity in Early Alzheimer’s Disease

**DOI:** 10.3390/ijms25021301

**Published:** 2024-01-21

**Authors:** Ahmed El Menuawy, Thomas Brüning, Iván Eiriz, Urs Hähnel, Frank Marthe, Luisa Möhle, Anna Maria Górska, Irene Santos-García, Helle Wangensteen, Jingyun Wu, Jens Pahnke

**Affiliations:** 1 Translational Neurodegeneration Research and Neuropathology Lab/Section of Neuropathology Research, Department of Pathology, Medical Faculty/KlinMED, University of Oslo (UiO) and Oslo University Hospital (OUS), Sognsvannsveien 20, 0372 Oslo, Norway; 2 Institute for Breeding Research on Horticultural Crops, Julius Kühn Institute (JKI)—Federal Research Centre for Cultivated Plants, Erwin-Baur Straße 27, 06484 Quedlinburg, Germany; 3 Section for Pharmaceutical Chemistry, Department of Pharmacy, University of Oslo (UiO), Sem Sælands vei 3, 0371 Oslo, Norway; 4 Institute of Nutritional Medicine (INUM) and Lübeck Institute of Dermatology (LIED), University of Lübeck (UzL) and University Medical Center Schleswig-Holstein (UKSH), Ratzeburger Allee 160, 23538 Lübeck, Germany; 5 Department of Pharmacology, Faculty of Medicine, University of Latvia, Jelgavas iela 3, 1004 Rīga, Latvia; 6 Department of Neurobiology, School of Neuroscience, Biochemistry and Biophysics, The Georg S. Wise Faculty of Life Sciences, Tel Aviv University, Tel Aviv 6997801, Israel

**Keywords:** *Hypericum perforatum*, St. John’s wort, Alzheimer’s disease, MCI, phytotherapy, silica gel, scCO_2_ extract, Syloid^®^ XDP3050

## Abstract

*Hypericum perforatum* (St. John’s wort) has been described to be beneficial for the treatment of Alzheimer’s disease (AD). Different extractions have demonstrated efficiency in mice and humans, esp. extracts with a low hypericin and hyperforin content to reduce side effects such as phototoxicity. In order to systematically elucidate the therapeutic effects of *H. perforatum* extracts with different polarities, APP-transgenic mice were treated with a total ethanol extract (TE), a polar extract obtained from TE, and an apolar supercritical CO_2_ (scCO_2_) extract. The scCO_2_ extract was formulated with silicon dioxide (SiO_2_) for better oral application. APP-transgenic mice were treated with several extracts (total, polar, apolar) at different concentrations. We established an early treatment paradigm from the age of 40 days until the age of 80 days, starting before the onset of cerebral β-amyloid (Aβ) deposition at 45 days of age. Their effects on intracerebral soluble and insoluble Aβ were analyzed using biochemical analyses. Our study confirms that the scCO_2_ *H. perforatum* formulation shows better biological activity against Aβ-related pathological effects than the TE or polar extracts. Clinically, the treatment resulted in a dose-dependent improvement in food intake with augmentation of the body weight, and, biochemically, it resulted in a significant reduction in both soluble and insoluble Aβ (−27% and −25%, respectively). We therefore recommend apolar *H. perforatum* extracts for the early oral treatment of patients with mild cognitive impairment or early AD.

## 1. Introduction

Alzheimer’s disease (AD) is a progressive neurodegenerative disorder characterized by depression, cognitive decline, memory loss, and functional impairment [1,2]. With the aging population worldwide, the prevalence of AD has been steadily increasing, posing a significant societal and healthcare challenge [3,4].

Amyloid-β (Aβ) has been shown to play a critical role in AD [5,6,7]. An elevated concentration of Aβ in the brain leads to insoluble Aβ deposits, also denoted as senile or amyloid plaques, resulting in synaptic dysfunction, neuroinflammation, and neuronal loss [8,9]. Aβ accumulation disrupts regular neuronal signaling pathways and impairs synaptic plasticity, ultimately causing the cognitive impairment and memory decline observed in the affected individuals [10]. Although Aβ is not the only biomarker, with TAU, for example, playing a significant role and showing better clinical correlations in patients with disease progression, the role of Aβ has been reaffirmed by the general effectiveness of monoclonal Aβ antibodies, despite their problematic side effects [11,12,13]. This substantiates the *Amyloid Hypothesis* [14,15], which posits Aβ as the primary trigger for the disease, while not negating the role of other biomarkers and their importance for disease progression and clinical disease severity [16,17], and thereby establishes Aβ as the main focus of our study.

Despite extensive research efforts, there is little to no effective curative treatment available [18]. The latest FDA-approved, disease-modifying monoclonal anti-Aβ antibodies (e.g., Adacanumab^®^, Lecanemab^®^) demonstrated a significant reduction in Aβ burden with a clinically minor delay in disease progression [19,20]. More importantly, extreme adverse effects occurred in up to 80.4% of the patients, referred to as Aβ-related imaging abnormalities (ARIA), including cerebral edema (ARIA-E, up to 30.7%) and cerebral hemorrhages (ARIA-H, up to 30.0%) (meta-analysis of 19 studies in [21]), and, thus, cause substantial concern about its use [19,20,22,23,24]. Subsequently, the identification of new therapeutic options targeting distinct mechanisms of action is of the highest interest [9,25,26].

ABCC1, also known as multidrug resistance-associated protein 1 (MRP1), belongs to the ATP-binding cassette (ABC) transporter superfamily. It is primarily involved in the efflux of a wide range of endogenous and xenobiotic substances, including glutathione, from cells. Beyond its role in drug resistance, ABCC1 has been implicated in neuroprotection, the modulation of inflammation, and the clearance of neurotoxic substances [27]. Previous research identified ABCC1 induction as a potential mechanism against Aβ-related, pathological brain effects [28,29,30].

*Hypericum perforatum* is a medicinal plant of the *Hypericaceae* family, native to Europe and traditionally widely used as an antidepressant [31,32]. Previous work has also shown the bioactivity of *H. perforatum* extracts with low hyperforin contents in AD model mice, resulting in a reduction of Aβ in brain tissue [28]. Building upon the findings that *H. perforatum* extracts with low hyperforin content show bioactivity in AD mouse models, leading to a reduction of Aβ in brain tissue, and demonstrate a hyperforin-independent activation of ABCB1 and ABCC1 transporters [17]—already identified as potential treatment targets for AD [5,14,15,19,22]—other studies have broadened this perspective. These investigations have identified additional compounds within the phloroglucinol group, such as hyperforone, as active substances [33]. Given the multifaceted nature of plant extract effects, which result from the complex interactions between numerous constituents [34], our research adopts a comprehensive approach. We are dedicated to unravelling the intricate details behind these varying results, with the goal of enhancing our understanding and pinpointing the most effective groups of active ingredients. This strategy aims at reconciling conflicting findings and honing in on the specific constituents that hold the most promise for AD treatment.

In addition to ABCC1, the activation of the adenosine triphosphate-binding cassette transport protein P-glycoprotein (ABCB1) has also been observed [35]. *Hypericum perforatum* has been shown to activate at least two transporters [28,35] that play a role in the clearance of toxic molecules from the central nervous system (CNS) [36]. This suggests a multitarget activation of ABC transporters by *H. perforatum*, indicative of its potential broad-spectrum therapeutic impact. The concerted action of these transporters, particularly in the context of neuroprotection and neurotoxic substance clearance, underscores the complex yet promising nature of *H. perforatum* in therapeutic applications.

To further develop effective and controlled AD therapy options based on *H. perforatum*, it is necessary to identify the extracts with optimal activities and reduced side effects. For this purpose, we obtained a total ethanol extract (TE), a polar extract obtained from the TE, and an apolar supercritical CO_2_ (scCO_2_) formulation, and tested the resulting fractions in an APP-transgenic (APPtg) mouse model. The apolar scCO_2_ formulation was identified as having optimal activity and an adapted treatment paradigm was applied to demonstrate its efficacy to treat Aβ-related pathology *in vivo*. To further elucidate the chemical composition of the plant extract, detailed analyses were conducted using ^1^H Nuclear Magnetic Resonance (NMR) and Heteronuclear Single Quantum Coherence (HSQC) spectroscopy, serving as primary methods for characterizing the extract’s molecular constituents [37,38].

## 2. Results 

Due to conflicting statements in the literature, a clear identification of the anti-dementia compounds from *H. perforatum* that work in AD animal models has not yet been achieved.

This study was conducted to clearly delineate active compounds from *H. perforatum* and to identify an effective extract. For this purpose, *H. perforatum* extracts with different polarities were used as an oral treatment in APPtg mice. Appropriate treatment regimens and concentrations were pre-tested and, finally, an early treatment paradigm [39] was employed with a duration of 40 days from 40 days of age. After treatment, protein brain extracts were fractionated to evaluate the treatment’s effects on the content of soluble and insoluble Aβ. Immunoassay determination of Aβ content from the fractions was then performed to evaluate the treatment efficacy of the herbal extract fractions.

The results showed that one formulation containing the apolar constituents (APOL) particularly resulted in an increase in body weight and increased activity of the animals, in contrast to the control group.

It is important to note that when the dosage of 3.30 mg/gBW was initially administered in the APOL group (protocol in Section 4.4.1.), adverse effects such as weight loss and signs of discomfort were observed (2/8 animals in TE group, 4/8 animals in APOL group). To ensure compliance with animal welfare guidelines, the affected mice were euthanized via cervical dislocation. To address this issue further, a study was conducted in which the full dose was replaced by a three-day dose titration (protocol in Section 4.4.2.). This completely eliminated the adverse effects.

In the final experiment, using dose titration and an adapted early treatment paradigm (protocol in Section 4.4.3.), a significant increase in body weight was again observed in two APOL-treated groups. In addition, one group showed a significant increase in food consumption per mouse. In particular, a reduction in the soluble and insoluble Aβ42 fractions in the brain was observed compared to the control group.

### 2.1. Formulation and Characterization of the APOL Extract

**Figure 1:**
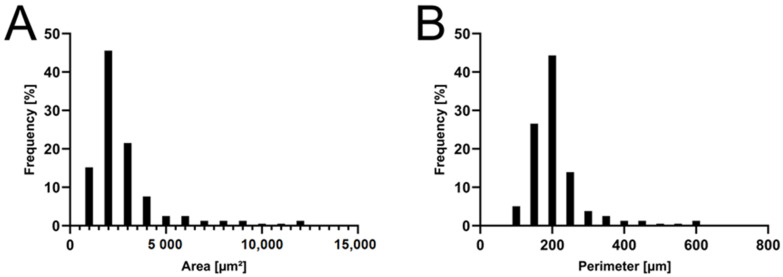
Distribution of APOL particles. Histogram of particle surface area (**A**) and particle circumference (**B**) of the APOL batch used. The distribution shows a right skew and thus does not follow a Gaussian normal distribution.

A commercially available supercritical CO_2_ extract from *H. perforatum* was chosen to test the apolar extract of *H. perforatum*. The oily and highly viscous properties of the extract presented a challenge for its oral administration via oral gavage. Therefore, a formulation with SiO2 was employed to overcome this obstacle. The result was a free-flowing yellow powder, suspendable in aqueous phase but not soluble. The powder is satisfactorily homogeneous in particle size, with 84.8% of particles in the range 150–250 µm in circumference and 82.3% of particles in the range 1000–3000 µm² in surface area (Figure 1). Inaccuracies in suspension delivery due to excessive differences in particle size are therefore negligible. The right skew of the histograms indicates a quality hurdle in production. It is likely that large particles result from the agglomeration of smaller particles due to cohesive properties of the scCO_2_ extract of *H. perforatum* during the manufacturing process. This could be avoided in the future by optimizing the manufacturing parameters. Adaptation of the manufacturing process is important for future industrial applications, but can be neglected for our small-scale study.

### 2.2. Toxicity Observations and Treatment Adjustments

Twenty-four hours following the initial oral administration, indications of adverse side effects were detected in the groups subjected to the total extract (“TE”) and CO_2_ extract treatments (“APOL”). Within the TE group (*n* = 8), two mice, and in the APOL group (*n* = 8), four mice, displayed adverse effects (apathy). Subsequently, the affected mice were euthanized, enabling symptom-free animals to continue treatment in the subsequent experiment at a reduced dosage without further incidents. Notably, no adverse symptoms were observed in the polar fraction (“POL”) and control groups. These events highlight that unspecified apolar substances, when administered at a high initial dose, as described in Section 2.4.1., can exert a toxic impact on mice.

**Figure 2:**
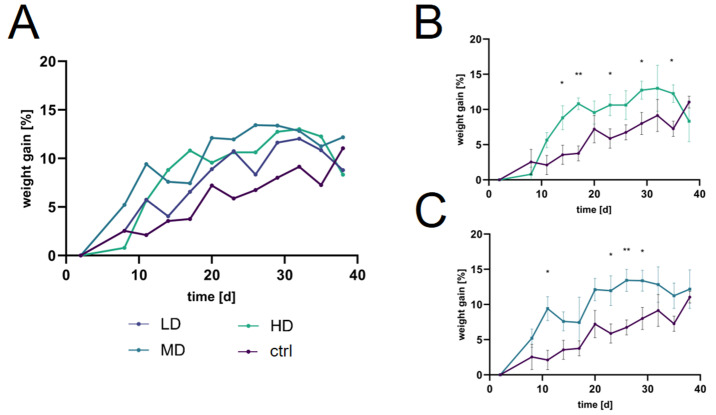
Weight gain of APOL extract-treated mice. (**A**) Relative increase [%] in body mass of mice treated with APOL over time across three distinct dosage levels. Direct comparisons between ‘HD’ (**B**) and ‘MD’ (**C**) treatments versus the control group (ctrl) reveals significant, dose-dependent effects. HD = high dosage, MD = medium dosage, LD = low dosage. Significant differences were assessed using Welch’s *t*-test, * indicates *p* < 0.05, ** *p* < 0.01.

The supercritical carbon dioxide (scCO_2_) extract contains approximately 49.0% hyperforins (Supplementary Material, File S1). Negres et al. [40] reported an LD50 value for hyperforin exceeding 5 mg/gBW, well above our maximum administered APOL dose of 3.3 mg/gBW. Therefore, it is probable that the toxicity arises from other constituents present in APOL or from potential interactions between these constituents. The precise toxic mechanisms associated with APOL require thorough investigation in future research. It is important to note that the observations were confined to the first 24 h following initial dosing. Given the absence of adverse effects such as pain or substantial weight loss (Figure 2), sustained exposure likely led to habituation effects. Consequently, dose titration appears to be the judicious approach.

### 2.3. Dosage strategy and Optimization

To avoid further adverse effects, dose titration was performed with the three different concentrations listed in Section 4.4.3 to determine a functional APOL dosing scheme. By reducing the initial dose, no adverse events occurred during the first 24 h. Furthermore, no apparent toxic effects were observed when the dose was gradually increased up to 24 h after the maximum dose on day 3.

In subsequent experiments, the dosage was escalated to achieve a maximum daily dosage of 1.8 mg per gram of body weight (mg/gBW) to maximize the desired effects while avoiding observable toxic side effects such as body weight loss. These results offer valuable insights for establishing dosage recommendations in future investigations involving APPtg and APOL.

**Figure 3:**
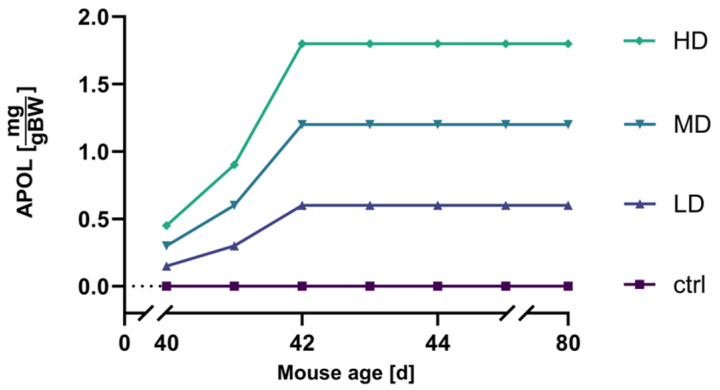
APOL dosage titration. Dosage scheme for three different concentrations of APOL per body weight, during the 40-day treatment period of mice. HD = high dosage, MD = medium dosage, LD = low dosage; control (ctrl) group were treated only with SyloidXDP3050^®^.

The best treatment efficiency was commenced starting at 40 days of age, prior to the onset of plaques, for a duration of 40 days (Figure 3). This temporal treatment regimen demonstrated the highest efficacy in terms of reducing Aβ42 levels, as outlined in Section 2.4.2.

### 2.4. Assessment of the Treatment Effects of H. perforatum Extracts

#### 2.4.1. Determination of Most Efficient Extract Fraction

**Figure 4:**
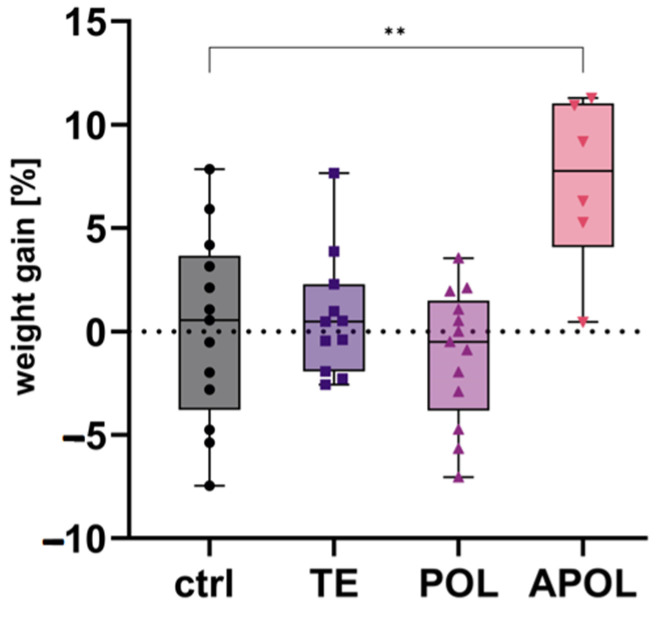
APOL treatment improved body weight. Shown is the relative body weight gain after 25 days of treatment with different fractions of *H. perforatum* (TE = total extract, POL = polar extract, and APOL = scCO_2_ extract from SyloidXDP3050^®^). The control group (ctrl) was treated with tap water. Pooled data of two repetitions are shown as box plots; *n* = 6–13; significant differences were assessed using Welch’s *t*-test, ** indicates *p* < 0.01, the symbols represent individual measurements.

To determine which fractions are responsible for the previously reported positive effects in AD treatment, fractions of different polarities were tested against a control group in two repetitions (protocol in Section 4.4.1). First, we detected a significant increase in body weight in the APOL group compared to the controls (Figure 4). No differences between the TE and POL groups and the control group were observed.

**Figure 5:**
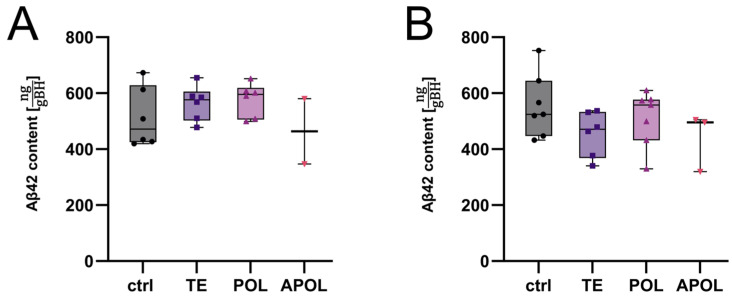
Aβ42 content in brain homogenates (BH) of animals treated with different fractions of *H. perforatum (*n_final_ = 2–7). First (**A**) and second (**B**) repetition of Aβ42 content quantification via immunoassay in the GuaHCl fraction of brain protein extracts from animals treated with different fractions of *H. perforatum* (TE = total extract, POL = polar extract, and APOL = scCO_2_ extract from SyloidXDP3050^®^). Control group (ctrl) was treated with tap water.

However, Aβ42 quantification did not reveal any significant differences compared to the control for any of the three extract fractions (Figure 5). Further reduced sample sizes were due to the exclusion of samples that did not meet quality standards during tissue harvesting or protein extraction.

#### 2.4.2. Further Exploration of the APOL Extract

To assess APOL, with the adjusted dosing regimen to prevent adverse effects, three different concentrations of APOL were tested (protocol Section 4.4.3). The findings highlight distinct trajectories for each dosage group (Figure 2A). Initially, the LD group displayed a moderate increase in body weight, which heightened rapidly and plateaued towards the end of treatment. The MD group maintained a steady incline in weight gain, characterized by minor fluctuations throughout the total observation period. The high-dosage (HD) group was particularly noteworthy, starting with a sharp rise, and, while it decelerated slightly over time, the trajectory remained elevated relative to both the LD and MD groups. The significant differences observed in the MD and HD groups, when compared to controls (Figure 2B,C), underscore the treatment-dependent effect on body weight.

**Figure 6:**
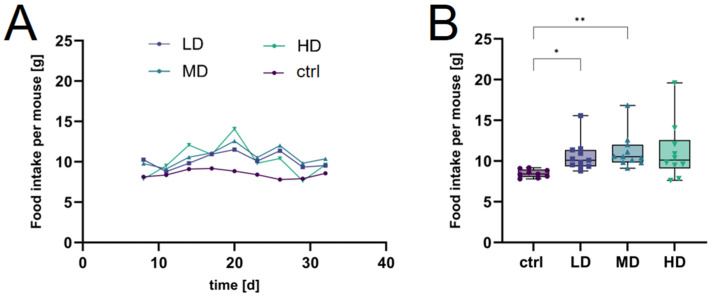
Food intake measurements during treatment. Change in daily food intake per mouse [g] treated with various dosages (LD, MD, and HD) over the treatment period (**A**) and average overall food consumption per mouse (**B**). Significant differences were assessed using Welch’s *t*-test; * indicates *p* < 0.05, ** *p* < 0.01, the symbols in (**B**) represent individual measurements.

The control group exhibited a consistent food intake across the treatment period, with only minor fluctuations (Figure 6A). The LD and MD groups’ intake showed slightly elevated intake rates in comparison to the controls, demonstrating minor variances. The HD group presented the most pronounced variation in food intake. The initial phase showed a marked increase in consumption compared to the control group. While there was a transient decline following this surge, intake levels for the HD group remained elevated throughout the study, exceeding that of the controls. However, no significant increase was found in the HD group due to the high variance, while the LD and MD groups showed a significant elevation of intake over the whole course of the treatment (Figure 6B).

**Figure 7:**
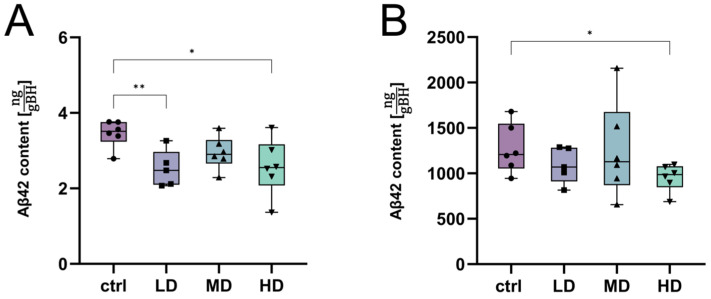
Aβ42 content in the soluble and insoluble protein fraction of APOL-treated mice. Aβ42 content in the TBS (**A**) and GuaHCl protein fractions (**B**) of the brain from APPtg mice treated with APOL at three concentrations (HD = high dosage, MD = medium dosage, LD = low dosage). Controls (ctrls) were treated with SyloidXDP3050^®^ only. Data are shown as box plots; *n* = 5–7; significant differences were assessed using Welch’s *t*-test: * indicates *p* < 0.05, ** *p* < 0.01, the symbols represent individual measurements.

Determination of Aβ42 content using the immunoassay of both protein fractions (GuaHCl and Tris-buffered solution) showed a significantly reduced Aβ load in the treated groups. A significant reduction in the amount of soluble Aβ42 in the TBS fraction per gram of homogenized brain tissue was observed in the LD and HD groups, with an average lower Aβ42 content of –26.98% for LD and –25.60% for HD in comparison to the ctrl group (Figure 7A). In the insoluble Aβ42 fraction, a significant reduction was observed in the HD group. The mean amount was reduced by –24.90% in the HD group compared to the control group (Figure 7B).

#### 2.4.3. Characterization of Major Extract Metabolites via NMR Spectroscopy

The ^1^H NMR and HSQC spectra of the APOL extract (scCO_2_) were dominated by hyperforin signals. This is in accordance with the certificate from the manufacturing company, which stated the a 48.5% presence of hyperforins. There were no signals from flavonoids or hypericin in the NMR spectra of the APOL extract. Characteristic signals from hyperforins are slightly deshielded signals from the methyl groups of the prenyl side chains, giving rise to intense signals in the area δ_H_ 1.5–1.8, which could be observed in the HSQC spectrum with corresponding carbon signals at δ_C_ 18–26. Other prominent signals from hyperforin were the signals from δ_H_ 4.9–5.2/δ_C_ 120–124, corresponding to the olefinic protons of the prenyl groups, methylene groups of the side chains δ_H_ 1.7–3.2/δ_C_ 22–30, and the shielded methyl groups at δ_H_ 1.0–1.1/δ_C_ 14–21 [41].

The ^1^H NMR spectra of the TE and POL extracts contained the same signals in the aromatic (5.8–8.0 ppm) and carbohydrate regions (3–5.5 ppm) of the spectra. Hyperoside was the major metabolite with characteristic aromatic signals at δ_H_ 7.83 (d), δ_H_ 7.58 (dd), δ_H_ 6.86 (d), δ_H_ 6.40 (d), and δ 6.20 (d). Epicatechin also demonstrated characteristic signals (e.g., δ_H_ 6.97 (d), δ_H_ 5.94 (d), and δ_H_ 5.91 (d)). We could also observe overlapping signals from other quercetin glycosides (δ_H_ 7.5–7.8, δ_H_ 6.8–6.9, and δ_H_ 6.2–6.4). The major difference between the TE and POL extracts was the absence of hyperforin signals in the POL extract. The prominent methyl signals in the area δ_H_ 1.5–1.8 in the total extract were totally missing in the POL extract [42,43] (Supplementary Material, File S2, Figures S1–S5).

## 3. Discussion

*Hypericum perforatum* is known as an efficient treatment against depression in elderly people, and is widely used in Europe [44]. Specific extracts that have proven their efficiency can also be prescribed by medical doctors and are paid for by national health systems, e.g., as LAIF900^®^ in Germany [45,46]. AD- and age-related depression often coincide with dementia or precede the symptoms of depression [1,47,48]. Thus, depression and dementia are two symptoms that are inevitably also connected biologically. It is therefore not surprising that specific plant extracts with anti-depressant characteristics could potentially be used as treatments for AD [30].

During many years of investigations into various plant extracts, we were able to specify lipophilic extracts of *H. perforatum* that possibly have better potential for interfering with dementia symptoms and their biological cause, namely Aβ deposition [28] or neuronal death [49]. 

NMR spectroscopy is a powerful technique for the fingerprinting of the major metabolites of herbal extracts, including *H. perforatum* [50]. One of the main advantages with NMR compared to HPLC is that no analysis of standard compounds is needed, as long as reference spectra are available. NMR showed that quercetin glycosides, with the major one being hyperoside, were the dominating aromatic molecules of the total and polar extracts, while hyperforins generated the major signals of the apolar (APOL) CO_2_ extract. The total and polar extracts also contained large amounts of carbohydrates. A prominent difference between the total (TE) and polar (POL) extracts was the absence of hyperforin signals in the polar extract.

Having faced technical problems in applying the extracts produced with supercritical CO_2_ to mice [28], we decided to solve this problem. The method presented here for formulating highly apolar extracts, such as APOL, with SiO2, enables their oral application for in vivo experiments with AD mouse models. Nevertheless, there remains room for further optimization of the formulation process, such as a refinement of the production methodology and particle size filtration to achieve a more homogeneous particle size distribution, thereby enhancing the precision in the suspension dosage’s administration.

Our initial experiments showed that TE and POL extracts have low to no efficiency in animal experiments at reducing the Aβ content in brain tissue. The APOL group could not be used to determine any effects of the extract on Aβ content due to the adverse effects experienced at the dosage we used. Side effects are also often discussed in patient treatment using *H. perforatum* [51]. While weight change is a non-specific parameter influenced by multifactorial aspects, it cannot be used to draw definitive conclusions on its own. However, it has previously been correlated with clinical progression in AD [52,53,54]. This observation, in conjunction with our ELISA findings (Section 2.4.2), leads us to propose that variations in weight and food intake might represent a secondary beneficial effect in mice. Specifically, weight maintenance or loss at this stage may be indicative of dysfunction, potentially linked to toxic or amyloid-mediated disturbances. Therefore, in the context of our comprehensive data analysis, the marked increase in body weight and food consumption in mice treated with APOL appears to exert a favorable impact on their overall condition. We observed a significant increase in food consumption after 20 days of the intervention, which finally converges with the control group. This phenomenon might be attributed to the animal’s adaptation to the bioactive compounds in the APOL extract, potentially via mechanisms like enhanced metabolism through liver cytochrome enzyme induction [55]. Such adaptation might also elucidate the augmented tolerance towards the extract’s toxicity following the initial dosing [56]. To elucidate these observations further, additional studies need to be performed to define the optimal therapeutic setting.

We therefore employed a series of APOL investigations to titrate the toxicity in mice before we continued. In the final experiment, with three adapted APOL dosages, we detected two treatment groups that had significantly increased food intake and body weight. This again allowed for the conclusion that the increased body weight is partly caused by improved feeding behavior. Additionally, we detected a reduction in Aβ42 levels in both protein extraction fractions: soluble and insoluble Aβ, respectively. We therefore concluded that the apolar constituents derived from *H. perforatum* exhibit the best effect to reduce the Aβ42 burden in brains of APPtg mice. Former investigations by our group have suggested that this mitigation could potentially be caused by an improvement in the soluble Aβ clearance facilitated by the activation of ABC transporters, in particular, ABCC1 [28,30]. The reduced concentration of soluble Aβ42 within the brain of treated mice leads further to a reduction in insoluble Aβ (aggregates/plaques). Both soluble and insoluble Aβ contribute to the total toxicity of Aβ peptides in the brain, and the removal of the soluble Aβ before touching the insoluble aggregates [9,25,26] might reduce the side effects caused by structural problems in vessels (due to congophil amyloid angiopathy [57,58]) seen in current treatments that use anti-Aβ antibodies, such as ARIA, ARIA-E, and ARIA-H [21,22].

## 4. Materials and Methods

### 4.1. Animal Models and Breeding Scheme

Animal model maintenance and husbandry were performed as previously described [39,59,60]. The individuals used in this study were housed at the Department of Comparative Medicine (section of the Radium Hospital) at the Oslo University Hospital (Oslo, Norway). In this facility, the room macro-environmental parameters included a relative humidity of 62 ± 5%, 15 air changes per hour, an average temperature of 22 ± 1 degree Celsius, and light cycles of 12 h dark/12 h light (strength: 1 lux at night, 70 lux during the day, 400 lux for working illumination). Health monitoring was performed thrice a year according to FELASA guidelines, including opportunists in one of the tests as well. In our experiments, mice were grouped into cohorts of 6–7 individuals in Eurostandard type III cages (Makrolone^®^), provided aspen wood (*Populus tremula*, Tapvei^®^, Paekna, Estonia) as bedding substrate, and offered additional enrichment material (tissue paper, tunnel rods, and, occasionally, gnawing sticks). They were fed ad libitum (Rat and Mouse No. 1 Maintenance expanded pellets from SDS, Estonia) and their water was acidified to pH 3 to limit bacterial growth. All experiments were conducted in accordance with the guidelines for animal experiments of the European Union directive and national laws.

The models used in this study were female APPPS1-21 mice [61] (B6.Cg-Tg(Thy1-APPSw,Thy1-PSEN1*L166P)/21JkcrPahnk, APPtg). APPPS1-21 mice have a combined *APP* (Swedish mutations) and *PS1* (L166P mutation) transgene under the control of the *Thy1*-promoter, leading primarily to pathological Aβ production in the fronto-cortical neurons and their first cortical Aβ plaques at 45–50 days of age, which also occurs much later in other brain regions as well, but to a significantly lesser extent (e.g., the hippocampus) [29,62].

### 4.2. Production of Plant Material

Seeds of *H. perforatum* were procured from the seed bank of the Julius-Kühn-Institute (Quedlinburg, Germany). Following germination in a climate chamber at 18 °C for 14 days, they underwent 12 weeks of greenhouse cultivation at 22 °C under artificial illumination provided by halogen vapor lamps in a short-day rhythm of 10 h daily. The resulting plants were transplanted into a randomized block design, with 10 individual plants per replication. Upon reaching maturity, the inflorescences were harvested and subjected to a three-day drying process in a drying chamber set at 30 °C. To ensure robust results, a composite sample was meticulously created by combining three technical replicates and subsequently utilized for extraction.

### 4.3. The Extraction and Formulation of H. perforatum 

Grounded *Hyperici herba* (Ph. Eur. 10.0, 1438 (12/2020)), weighing 200 g, was extracted with 1000 mL of 70% (*v*/*v*) ethanol using ultrasound-assisted extraction. The resulting ethanolic extract was taken to dryness by rotary evaporation followed by freeze-drying, resulting in the formation of a solid product with a characteristic purple crystalline appearance. This solid product was labelled as the dried total extract (TE).

The TE was suspended in water and defatted via liquid–liquid extraction, using heptane as the extraction solvent. After removal of the heptane fraction, the resulting aqueous fraction was subjected to the same drying procedures as mentioned above, resulting in the polar fraction (POL), a purple, crystalline, solid mass.

To encompass the apolar substances present in *H. perforatum*, a comprehensive fraction was obtained by procuring a commercially available supercritical CO_2_ (scCO_2_) extract from FLAVEX Naturextrakte GmbH (Lot nr.: 012501, Rehlingen, Germany).

The scCO_2_ extract was first dissolved in heptane, and then silicon dioxide (SiO_2_, Syloid^®^ XDP3050, Grace GmbH, Worms, Germany) was added [63,64,65]. The solvent mixture was subjected to complete evaporation under rotation and vacuum using a rotary evaporator (Hei-VAP Core HL G3B XL, Heidolph Instruments, Schwabach, Germany).

Following the evaporation process, heptane was removed and a yellow free-flowing powder was obtained. This powder exhibited a metastable suspension when introduced into an aqueous solution containing 1.5% methylcellulose. The resulting apolar suspension (APOL) was suitable for oral administration and facilitated the delivery of the scCO_2_ extract via oral gavage.

### 4.4. Treatment Schemes

To evaluate the effects of *H. perforatum* on the APPtg mice, several experiments were conducted, which are elaborated in the following sections. Initially, an assessment was performed using *H. perforatum* extracts of different polarities. Subsequently, an experiment was conducted to optimize the dosing regimen. Following this, a final experiment with an early treatment paradigm was carried out using the most promising dosing scheme, applying three different dosages across three distinct groups.

#### 4.4.1. Treatment Scheme to Test Hypericum Perforatum Extracts (TE, POL, and APOL)

Female mice were first treated with three different extracts of *H. perforatum* via oral gavage for a duration of 25 days (49 to 75 days of age) in three distinct groups of females (*n* = 8) and in two repetitions (Table 1).

Dried extracts and solid preparations were dissolved in either 1.5% methylcellulose (APOL) or tap water (TE, POL) through stirring for 1–2 h at room temperature (TE, POL) or vortexing for 1 min (APOL). Body weight was recorded weekly throughout the entire duration of treatment. The control groups received tap water. Mice initially received 4.0 mg/gBW TE and POL and 3.3 mg/gBW APOL, respectively. After first dose, the TE and APOL dose was reduced by 50% due to signs of toxicity, resulting in 2.0 mg/gBW for TE and 1.65 mg/gBW for APOL, respectively.

#### 4.4.2. Dosage Titration of the APOL Extract

For further elucidation of the optimal APOL dose titration, two separate groups of female APPtg mice were subjected to a three-day-treatment regimen. 

The initial administered dose was set to 0.300 mg/gBW (APOL**_1_**, *n* = 8) and 0.075 mg/gBW (APOL**_2_**, *n* = 8), respectively. Subsequently, a dosage escalation was carried out over the course of the second and third treatment by doubling the dose daily to a maximal dosage of 1.2 mg/gBW and 0.3 mg/gBW across the two delineated groups (Table 2).

#### 4.4.3. Early Treatment Paradigm to Assess Efficacy of the APOL Extract Fraction

In the subsequent phase of testing, female mice were treated with three different concentrations (*n* = 7) of APOL via oral gavage in an early treatment paradigm with a duration of 40 days, starting at 40 days of age and continuing until 80 days of age. The suspension was prepared using 1.5% methylcellulose and further vortexed for one minute. The control group received an equally suspended unloaded SyloidXDP3050^®^ in 1.5% methylcellulose. Body weight and food intake were recorded in 3-day intervals for the entire duration of treatment (Table 3).

### 4.5. Tissue Harvesting

Mice were euthanized via cervical dislocation. After intracardial perfusion with ice-cold PBS, their brains were removed and separated into two hemispheres. One hemisphere was kept in paraformaldehyde (PFA 4% in PBS, 48 h), the other hemisphere was snap frozen in liquid nitrogen and later transferred to −80 °C until further use.

### 4.6. Protein Extraction

Frozen hemispheres were thawed on ice in 500 µL RNAlater^®^ (Merck KGaA, Darmstadt, Germany) for one hour, removed from liquid and homogenized for 60 s with four 2.8 mm ceramic beads (OMNI International, Atlanta, GA, USA) using a SpeedMill PLUS (Analytik Jena GmbH, Jena, Germany). Twenty milligrams of homogenate was mixed with 10 µL of cold Tris-buffered saline (TBS, pH 7.5, containing Roche protease inhibitor (VWR, Oslo, Norway)) per 1 mg brain. Samples were homogenized with 2.8 mm ceramic beads (SpeedMill PLUS, 30 s) and centrifuged (16,000× *g*, 4 °C, 20 min) to separate soluble and insoluble Aβ. The resulting supernatant (TBS fraction containing soluble Aβ) was collected and stored at −20 °C until further use. The pellet was mixed with 8 µL cold 5 M Guanidine HCl buffer (pH 8.0) per 1 mg brain homogenate, and homogenized (SpeedMill PLUS, 30 s). Samples were incubated at room temperature for 3 h under constant shaking (1500 rpm) before centrifugation (16,000× *g*, 4 °C, 20 min). The supernatant (GuaHCl fraction originally containing insoluble Aβ) was collected and stored at −20 °C until further use.

### 4.7. Quantification of Aβ42

To quantify Aβ42 in TBS (soluble) and GuaHCl (insoluble) fractions, we performed electrochemiluminescence immunoassays using the V-PLEX Plus Aβ42 Peptide (4G8) Kit and a MESO QuickPlex SQ120 machine, according to manufacturer’s recommendations (MesoScale Diagnostics, Rockville, MD, USA) [66,67,68]. TBS fraction analysis was limited to experiments showing significant differences in Aβ42 in GuaHCl fractions.

### 4.8. NMR Spectroscopy of Extracts

^1^H NMR and HSQC spectra were recorded on a Bruker AVNEO400 instrument (Bruker, Rheinstetten, Germany). CD3OD was used as solvent for the total and polar extracts, and CDCl3 was used for the scCO_2_ extract.

### 4.9. Statictics

Statistical analysis was performed using Graphpad Prism software (v9, Dotmatics, Boston, MA, USA). We verified the data for Gaussian normal distribution using the Shapiro–Wilk normality test [69]. All data were cleaned of outliers by ROUT (Q = 1%). Welch t-tests were performed to determine the significant differences between two groups. Data are presented as means ± standard deviation (SD). Differences were considered statistically significant when *p* < 0.05. N is reported in the Figure legends.

## 5. Conclusions

Our study confirms that the scCO_2_ *H. perforatum* formulation shows the best biological activity against Aβ-related pathological effects. Therefore, we recommend apolar *H. perforatum* extracts for the early treatment of mild cognitive impairment or for early AD patients.

## Figures and Tables

**Table 1:** Treatment scheme for pre-testing of *H. perforatum* extracts. Mouse body weight-adjusted dosage (mg/gBW) for different extracts (TE = total extract, POL = polar extract, and APOL = scCO_2_ extract from SyloidXDP3050^®^). Treatment started at 49 days of age and ended at 75 days of age.

Mouse Age [d]	TE	POL	APOL
49	4.00	4.00	3.30
50	2.00	4.00	1.65
51	2.00	4.00	1.65
52–75	2.00	4.00	1.65

**Table 2:** Treatment scheme for dosage titration [mg/gBW] of APOL extracts (toxicity assessment) over three consecutive days, starting at an age of 49 days.

Mouse Age [d]	APOL_1_	APOL_2_
49	0.300	0.075
50	0.600	0.150
51	1.200	0.300

**Table 3:** Dosage scheme for three different concentrations (mg/gBW) of APOL per body weight, during the 40-day early treatment period of mice (HD = high dosage, MD = medium dosage, LD = low dosage, ctrl = controls treated with SyloidXDP3050^®^ only).

Mouse Age [d]		APOL		Ctrl
	HD	MD	LD	
39	0.0	0.0	0.0	0.0
40	0.600	0.150	0.075	0.600
41	0.900	0.300	0.150	0.900
42–80	1.800	0.600	0.300	1.800

## Data Availability

Data files and Figures can be downloaded from the ‘Pahnke Lab-open access’ project at DOI: 10.17605/OSF.IO/VWQ58.

## Supplementary Materials

The supporting information can be downloaded at https://www.mdpi.com/article/10.3390/ijms25021301/s1.

## Abbreviations

Aβ, amyloid-β; ABC transporter, ATP-binding cassette transporter; AD, Alzheimer’s disease; APP, β-amyloid precursor protein; ARIA, amyloid-related imaging abnormalities; GuaHCl, guanidine hydrochloride; mg/gBW—milligram per gramm of bodyweight; ng/gBH—nanogram per gramm homogenized brain; MW, molecular weight; PBS, phosphate-buffered saline; PFA, paraformaldehyde; SD, standard deviation; SiO_2_—silicon dioxide; TBS, TRIS-buffered saline.
