# Supplementary material for: Apolar Extracts of St. John’s Wort Alleviate the Effects of β-Amyloid Toxicity in Early Alzheimer’s Disease
Source: Int J Mol Sci. 2024 Jan 21;25(2):1301. doi: 10.3390/ijms25021301 (PMC10816143; doi:10.3390/ijms25021301)
Supplement: Supplementary file 1 [file ijms-25-01301-s001.zip › Supplementary Materials - File S1 _ APOL - Flavex_analytic certificate_CO2 extract.pdf]

931687-DE-3102

ECOCERT
COSMOS APPROVED

NATRUE Approved

Hochdruckextraktion mit CO_2

since 1986

FLAVEX®
Naturextrakte

Analysenzertifikat

Johanniskraut CO2-to Extrakt, Typ Nr. 028.001

Charge Nr. 012501, Labor Nr. 33044

Produktion: Dezember 2021	Retest: Dezember 2023
Rohstoff:	*Hypericum perforatum* - Zweigspitzen mit Blüten, getrocknet
Ursprungsland für Rohware:	Bulgarien
Ursprungsland für Produkt:	Deutschland
D/E - Verhältnis:	14 - 25 kg Rohstoff ergeben 1 kg Produkt.
Produkt enthält:	keine Träger- und Zusatzstoffe

Sensorische Prüfung

Merkmal	Anforderung	Befund
Aussehen:	hell bis dunkelbrauner, bei 50°C zähfließender Extrakt	entspricht
Geruch:	charakteristisch	entspricht

Analytische Prüfung

Merkmal	Methode	Sollwert	Wert	Einheit
Hyperforin	21.091.05, HPLC	n.s.	40,1	%
Adhyperforin	21.091.05, HPLC	n.s.	8,4	%
Gesamthyperforin	21.091.05, HPLC	30 - 50	48,5	%
Trocknungsverlust	Ph. Eur. 2.2.32 (2 h 105°C)	< 15,0	10,9	%
Hypericin	21.091.05, HPLC	n.d.	n.d.	%
Pestizide	externe Analyse	EC No. 396/2005	entspricht	

n.s. = nicht spezifiziert n.d. = nicht detektierbar

Das Produkt entspricht der aktuellen Version der analytischen (028.001_05_AS) und der allgemeinen (028.001_09_S) Spezifikation.
Analysendatum: 15.12.2021
Dieses rechnergestützte AZ ist digital signiert und durch die FLAVEX QC bestätigt.

Digital signiert von Anja Cawelius
Grund: Qualitätskontrolle
Datum: 2022.03.16 12 54:44+01'00'

Die Analysenwerte dieses Zertifikates wurden sorgfältig und nach unserem besten Wissen ermittelt. Je nach Transport- und Lagerbedingungen können die angegebenen Werte gewissen Schwankungen unterliegen, auf die wir keinen Einfluß haben. Daher ist das Zertifikat rechtlich unverbindlich und befreit den Kunden nicht, vor Anwendung des Produkts eine eigene Qualitätskontrolle durchzuführen.

FLAVEX Naturextrakte GmbH Fon +49 (0)6835 91950 E-Mail: info@flavex.com
Nordstr. 7, D 66780 Rehlingen Fax +49 (0)6835 919595 http://www.flavex.com
