# Supplementary material for: Apolar Extracts of St. John’s Wort Alleviate the Effects of β-Amyloid Toxicity in Early Alzheimer’s Disease
Source: Int J Mol Sci. 2024 Jan 21;25(2):1301. doi: 10.3390/ijms25021301 (PMC10816143; doi:10.3390/ijms25021301)
Supplement: Supplementary file 1 [file ijms-25-01301-s001.zip › Supplementary Materials - FIle S2 _ NMR.pdf]

Supplementary Material for the article

Apolar extracts of St. John's wort alleviate effects of β-amyloid toxicity in early Alzheimer's disease

Ahmed El Menuawy *et al.*

PUBL INFO HERE IJMS 2024 DOI

Supplementary Material / File S2 - NMR analyses

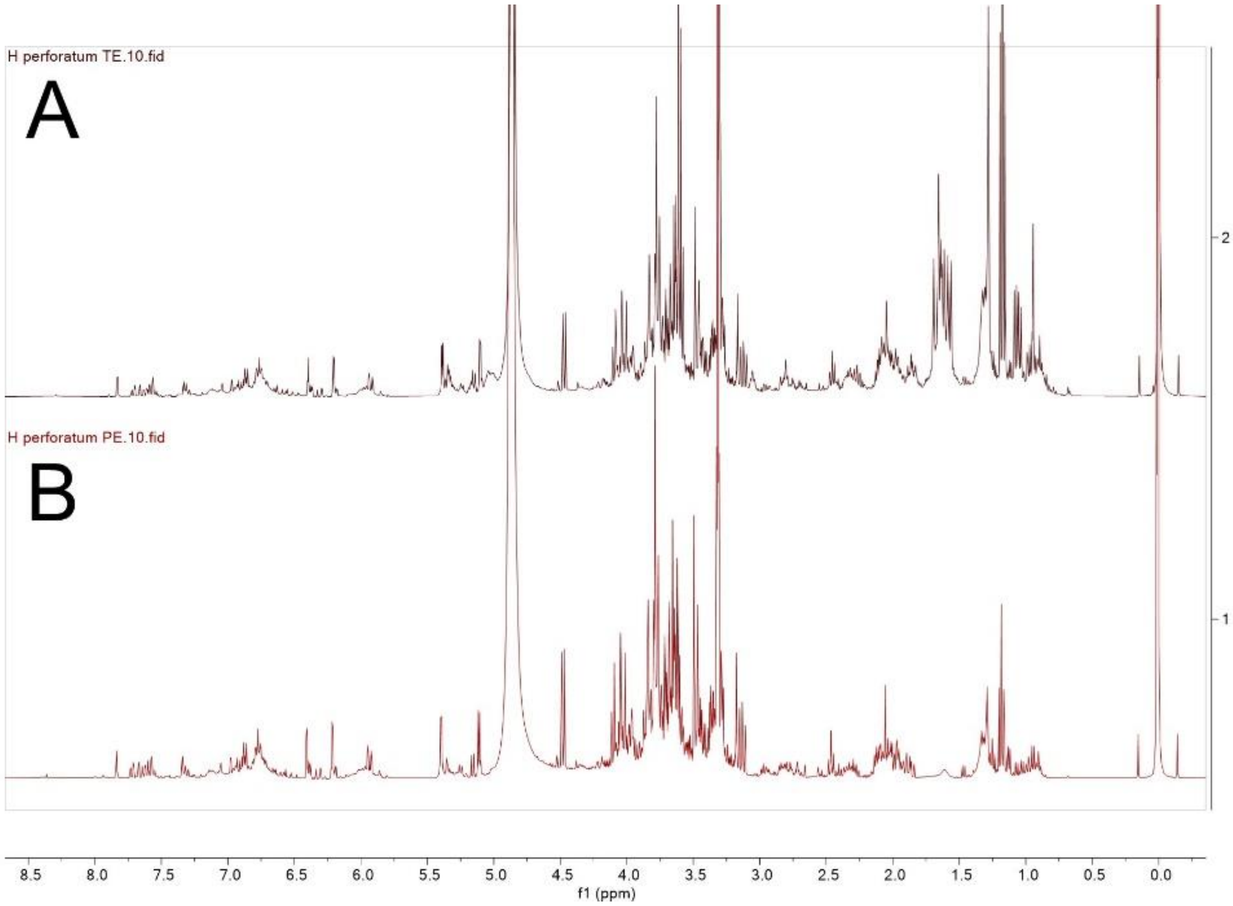

Supplementary Figure 1. Stacked ^{1}H NMR spectra of total extract (TE) (A) and polar extract (POL) (B).

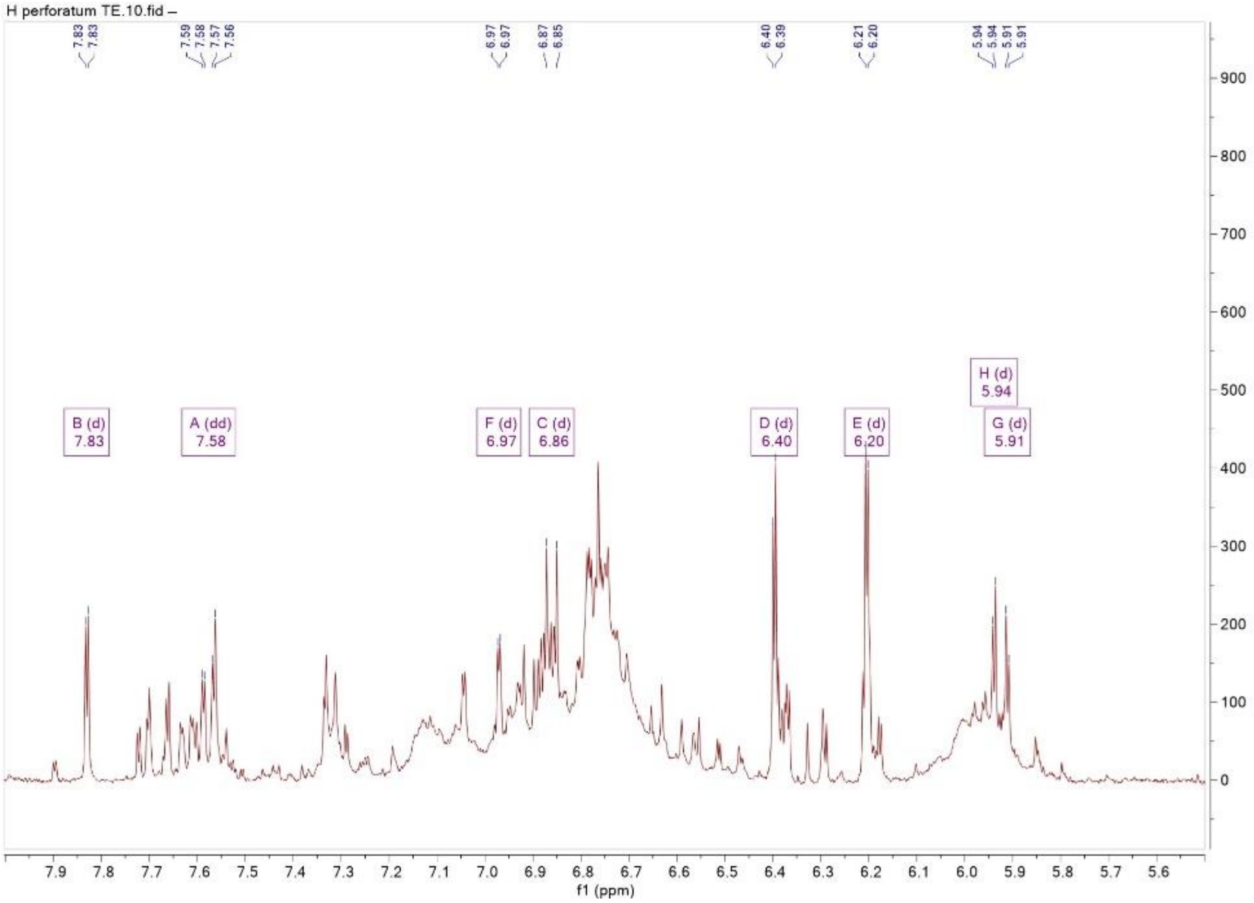

Supplementary Figure 2. Aromatic region of the ^{1}H NMR spectrum of TE.

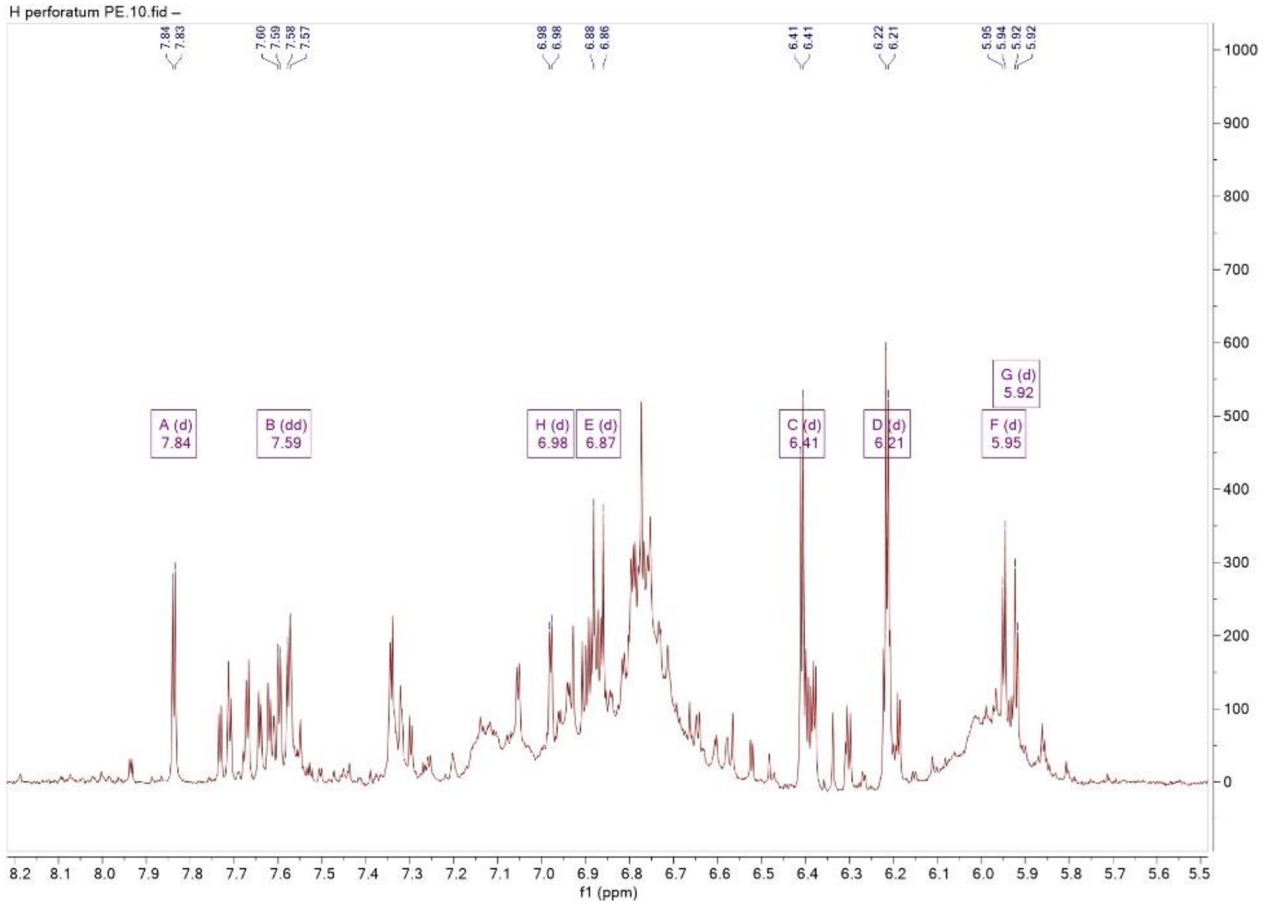

Supplementary Figure 3. Aromatic region of the ^{1}H NMR spectrum of POL.

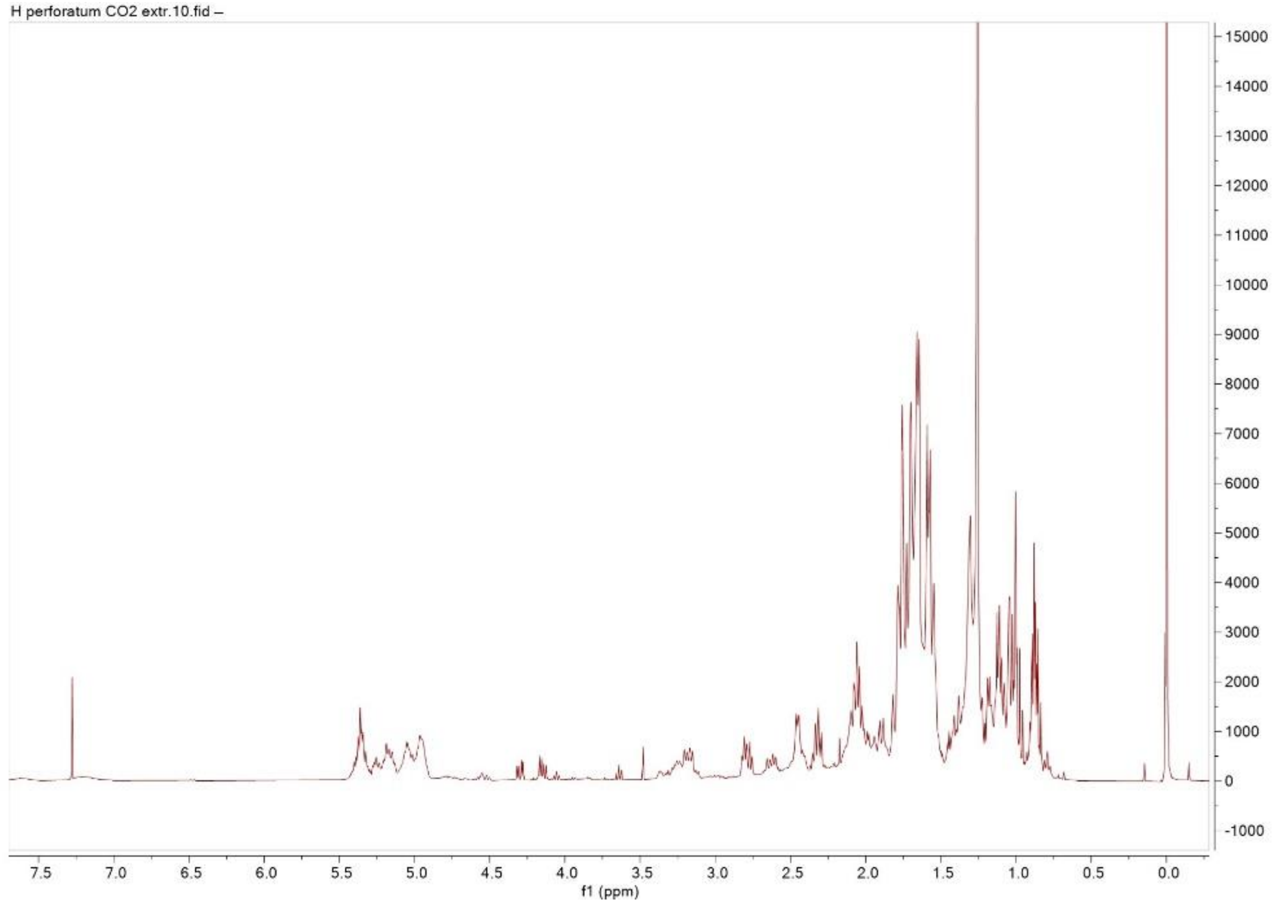

Supplementary Figure 4. 1H NMR spectrum of scCO2 extract (APOL).

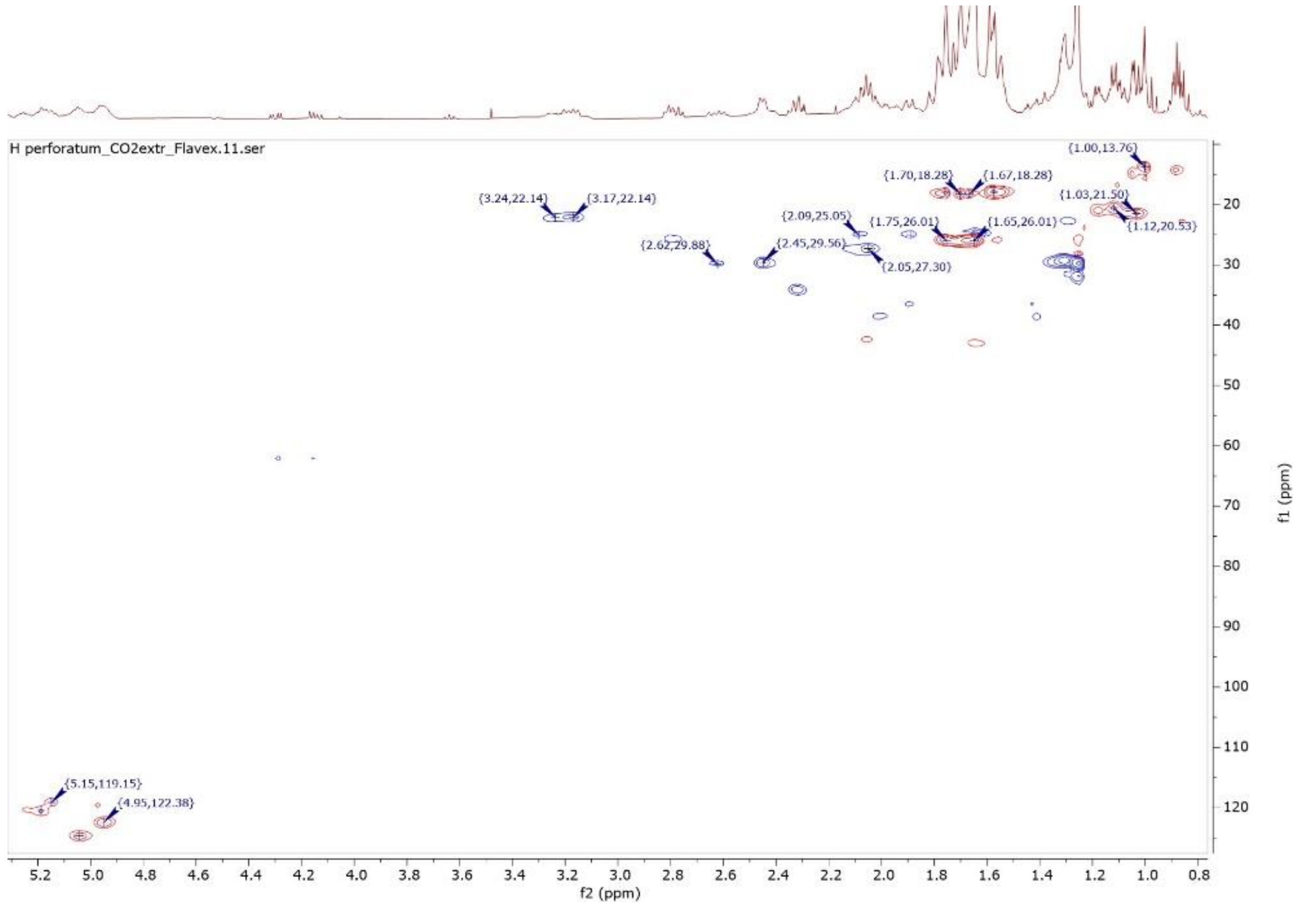

Supplementary Figure 5. HSQC spectrum of APOL ectract, signals from hyperforin are shown.
